# Applying a novel kinomics approach to study decidualization and the effects of antigestagens using a canine model^†^

**DOI:** 10.1093/biolre/ioad170

**Published:** 2023-12-11

**Authors:** Isabelle De Geyter, Mariusz P Kowalewski, Miguel Tavares Pereira

**Affiliations:** Institute of Veterinary Anatomy, Vetsuisse Faculty, University of Zurich, Zurich, Switzerland; Institute of Veterinary Anatomy, Vetsuisse Faculty, University of Zurich, Zurich, Switzerland; Center for Clinical Studies, Vetsuisse Faculty, University of Zurich, Zurich, Switzerland; Institute of Veterinary Anatomy, Vetsuisse Faculty, University of Zurich, Zurich, Switzerland

**Keywords:** decidualization, kinomics, dog uterine stromal (DUS) cells, progesterone, antigestagens, serine/threonine kinases, PKA, PKC

## Abstract

Maternal decidual cells are crucial for the maintenance of canine pregnancy as they are the only cells expressing the nuclear progesterone (P4) receptor (PGR) in the placenta. Interfering with P4/PGR signaling adversely affects decidual cells and terminates pregnancy. Although immortalized dog uterine stromal (DUS) cells can be decidualized in vitro using cAMP, the involvement of cAMP-dependent kinases in canine decidualization had not been investigated. Therefore, the present project investigated changes in the kinome of DUS cells following in vitro decidualization, using the serine/threonine kinase (STK) PamChip assay (PamGene). Decidualization led to a predicted activation of 85 STKs in DUS cells, including protein kinase (PK) A, PKC, extracellular signal-regulated kinase (ERK)1/2 and other mitogen-activated protein kinases (MAPKs), calcium/calmodulin-dependent protein kinases (CAMKs), and Akt1/2. In addition, blocking PGR with type 2 antigestagens (aglepristone or mifepristone) decreased the activity of virtually all kinases modulated by decidualization. The underlying transcriptional effects were inferred from comparison with available transcriptomic data on antigestagen-mediated effects in DUS cells. In targeted studies, interfering with PKA or MAPK kinase (MEK)1/2 resulted in downregulation of important decidualization markers (e.g., insulin-like growth factor 1 (*IGF1*), prostaglandin E2 synthase (*PTGES*), prolactin receptor (*PRLR*), *PGR*, and prostaglandin-endoperoxide synthase 2 (*PTGS2*/*COX2*)). Conversely, blocking of PKC decreased the mRNA availability of *IGF1*, *PGR*, and *PTGS2*, but not of *PTGES* and *PRLR*. Moreover, suppressing PKA decreased the phosphorylation of the transcription factors cJUN and CREB, whereas blocking of PKC affected only cJUN. This first kinomics analysis to target decidualization showed an increased activity of a wide range of STKs, which could be hindered by disrupting P4/PGR signaling. Decidualization appears to be regulated in a kinase-dependent manner, with PKA and PKC evoking different effects.

## Teaser

Serine/threonine kinases in canine uterine stromal cells increase during decidualization but are disrupted by P4/PGR signaling interference; PKA and PKC have distinct effects on decidualized cells’ function.

## Graphical Abstract

**Graphical Abstract:**
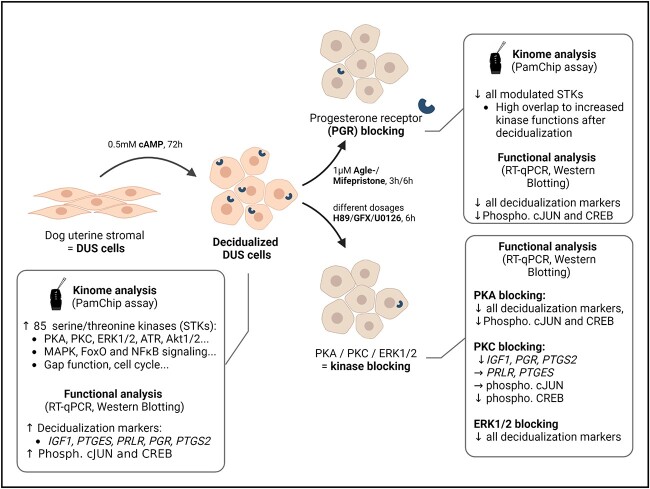

## Introduction

The establishment of pregnancy relies on complex interactions between the conceptus and maternal uterine tissues associated with precise morphological and biochemical processes, critical for creating an optimal uterine environment for embryo development. In species with invasive placentation (hemochorial and endotheliochorial), this interaction with the embryo involves the differentiation of endometrial stromal cells into decidual cells [1–3], which play a crucial role in controlling maternal local immunity, regulating trophoblast invasion, and nourishing the developing embryo [2–4]. In contrast to the hemochorial placenta, in which decidual cells form a decidua tissue layer [1, 2], the decidual cells are localized between maternal capillaries and fetal trophoblast in the domestic dog, which has restricted trophoblast invasion characteristic of the endotheliochorial placenta [1, 3]. In addition, the canine reproductive cycle has unique characteristics that limit the application of translational aspects from other species with invasive placentation. Most notable is the lack of placental steroidogenic activity [5–8]. Yet, despite the high levels of circulating luteal P4, spontaneous decidualization is not observed in non-pregnant animals, as decidualization is induced by embryo attachment [9]. The development of decidual cells and associated morphological changes are accompanied by the increased expression of decidua-specific factors (decidualization markers), including prostaglandin E2 synthase (PTGES), insulin-like growth factor 1 (IGF1), and various hormonal receptors such as the nuclear progesterone (P4) receptor (PGR), estrogen receptor alpha (ERα), and oxytocin receptor (OXTR) [9–11].

The diminishing or disrupted PGR signaling ultimately terminates pregnancy and leads to parturition or abortion if antigestagens (competitive PGR blockers) are applied [12]. Consequently, the type II antigestagen aglepristone remains the treatment of choice when inducing parturition/abortion in dogs [13]. In this regard, the role of decidual cells in canine pregnancy is pivotal, as they are the only placental cell population expressing the PGR [14, 15]. Therefore, studying the morpho-functional properties of decidual cells is crucial for understanding the complex mechanisms that regulate canine pregnancy.

Important insights into the mechanisms associated with the differentiation and function of canine decidual cells have been gained through the development and application of immortalized dog uterine stromal (DUS) cells [16]. Universally, in a decidual placenta, including humans, the process of decidualization is associated with elevated intracellular levels of cyclic adenosine monophosphate (cAMP), with signaling through the cAMP-dependent protein kinase (PKA) pathway [17]. Similarly, DUS cells respond to cAMP by going through a mesenchymal–epithelial transition and exhibiting an increased expression of decidualization markers, e.g., insulin-like growth factor 1 (*IGF1*), prostaglandin E2 (*PGE2*) synthase (*PTGES*), prolactin receptor (*PRLR*), *PGR*, and prostaglandin-endoperoxide synthase 2 (*PTGS2/COX2*) [16, 18–20]. Furthermore, as revealed via a transcriptomic approach, in vitro decidualization is associated with the modulation of the extracellular matrix and cell adhesion [21], as well as with cellular proliferation and migration, vasomodulation, and immune regulation [18, 19, 21].

P4 and PGE2 have been shown to have reciprocal activity in regulating the decidualization process [20]. Thus, whereas PGE2 increases the expression of PGR, blocking the functionality of cAMP-mediating PGE2 receptors, PTGER2 and -4, suppresses PRLR and PGR expression in decidualized DUS cells [20]. Similarly, when used simultaneously, P4 can enhance the decidualizing capacity of PGE2 by increasing the availability of PTGER2 [20].

Together with mifepristone, aglepristone is a type II antigestagen (reviewed in [13]). These compounds activate PGR while simultaneously disrupting its downstream signaling by recruiting co-repressors to the promoter region of target genes [22, 23]. In decidualized DUS cells, both antigestagens disrupt the expression of a wide range of factors including decidualization markers [19, 21]. Moreover, the viability of DUS cells also appeared to be affected by PGR inhibition [19].

Strikingly, however, despite the broad range of biological effects of decidual cells and the cAMP dependence of decidualization, the intracellular mechanisms active in decidual cells downstream of cAMP have never been assessed in the dog. In addition, the information available for other species appears incomplete. The same applies to the antigestagen-mediated effects, an understanding of which is of utmost clinical importance.

This project is based on the hypothesis that PKA and other serine/threonine kinases (STKs) are among the cellular responders to cAMP involved in canine decidualization. Thus, the aim of the present study was to assess the kinomic changes in the activity of STKs in DUS cells after decidualization and in response to antigestagens, using the PamChip assay. This is the first time this technology has been applied to the canine species, as well as to decidual cells of any mammalian species. Following observations from the kinomics analysis, the role of selected STKs in DUS cells was further dissected with targeted studies.

## Materials and methods

### Cell culture and in vitro experiments

All cell culture experiments were performed using the immortalized DUS cell line [16], with the procedures for cell handling following previously published protocols [16, 18, 19]. In brief, cells were cultured in a maintenance medium consisting of DMEM-High Glucose (Bio Concept, Allschwil, Switzerland), supplemented with 10% heat-inactivated fetal bovine serum (FBS; Thermo Fisher Scientific AG, Reinach, Switzerland), 100 U/mL penicillin and 100 μg/mL streptomycin (PAN Biotech, Aidenbach, Germany), and 1% insulin-transferrin-selenium (ITS; Gibco, Thermo Fisher Scientific AG). Cells were kept in 150-cm^2^ cell culture flasks (Corning, New York, NY, USA) in a humidified incubator under standard culture conditions (37 °C with 5% CO_2_ in air) until reaching at least 90% confluency. Cells were then trypsinized and 2.5 × 10^5^ cells were seeded per well into six-well plates (Corning), where they remained for 24 h in maintenance medium to recover from trypsinization and to allow attachment.

For the kinomics analysis, the following four experimental groups were used: control, DUS cells decidualized with cAMP, and decidualized cells treated with antigestagens (aglepristone or mifepristone) for 3 or 6 h. The cAMP-mediated decidualization was induced for 72 h in FBS-serum free medium containing 0.01% bovine serum albumin (BSA; SUB001, Canvax Biotech, Córdoba, Spain), i.e., stimulation medium, with 0.5 mM N6,2′-*O*-dibutyryladenosine-3′,5′-cyclicmonophosphate (dbcAMP, D0627; Sigma-Aldrich Chemie GmbH, Buchs, Switzerland). As the control group for all experiments, non-decidualized DUS cells were incubated in stimulation medium without further additives for the same length of time. For the antigestagen-treated groups, cells decidualized with cAMP for 72 h were subsequently treated either with mifepristone (RU483; Sigma-Aldrich Chemie GmbH) or aglepristone (RU534, Batch No: 2064665, kindly provided by Virbac, Tierarzneimittel GmbH, 23843 Bad Oldesloe, Germany) at a concentration of 1 μM for 3 or 6 h, as previously published [19, 21].

Finally, experiments targeting the activity of selected kinases were performed for 6 h and included the following groups: control, decidualized DUS cells (cAMP for 72 h), antigestagen-treated decidualized DUS cells, and decidualized cells treated with specific kinases inhibitors (three different dosages per blocker). Following the similarities of effects induced by both type II antigestagens, only mifepristone at 1 μM was used for these experiments (due to its commercial availability). Treatments with specific inhibitors targeted PKA (H89), PKC (GF-109203X/GFX), and MEK1/2 (U0126) (all reagents purchased from Sigma-Aldrich Chemie GmbH). Also, H89 and GF-109203X were administered at concentrations of 2.5, 5, and 10 μM, whereas U0126 was applied at concentrations of 1.25, 2.5, and 5 μM. All these concentrations were selected based on the literature, adjusted during preliminary experiments, and remained within the previously reported ranges [24–28]. Each experiment was repeated at least three times using cells derived from different passages.

### Lysate preparation and analysis of cellular kinomics activity

To perform the kinomics analysis, six experimental groups with cells from four consecutive passages (accounting for biological replicates) were used: non-decidualized/control (C); decidualized (cAMP); decidualized and treated with aglepristone (Agle) or mifepristone (Mife) for 3 or 6 h. At the end of treatment, cells were washed twice with ice-cold PBS and collected by scraping on ice in 100 μL M-PER Mammalian Protein Extraction Reagent containing Halt Phosphatase Inhibitor Cocktail and Halt Protease Inhibitor Cocktail EDTA-free, both diluted at 1:100 (all from Thermo Fisher Scientific AG). The cell lysate was homogenized by pipetting, transferred to pre-cooled vials, and centrifuged at 21500 g for 15 min at 4 °C. The supernatant was then collected and stored at −80 °C until use.

Protein concentrations in the lysates were quantified via the Bradford assay in a SmartSpec Plus spectrophotometer (Bio-Rad Laboratories, Munich, Germany), and normalized to 0.1 μg/mL with the addition of M-PER Mammalian Extraction Buffer. The activity of STKs was measured by flow-through microarray with STK PamChip Arrays on a PamStation 12 system (PamGene International BV, Hertogenbosh, The Netherlands). The STK PamChip array contains 144 immobilized substrates of STK, i.e., synthesized phosphorylatable peptides (13 amino acids long) attached to a porous membrane. Reagents and samples were pumped through the porous arrays in several cycles by the PamStation. Sample preparation and assays were performed following the protocol and with reagents provided by the manufacturer (PamGene International BV). Arrays were initially blocked with 2% BSA (30 cycles). Then, a total of 10 μL of lysate (equivalent to 1 μg of proteins) was added to the assay mixture containing primary STK antibody solution and 400 μM of ATP (PamGene International BV), and pumped (60 cycles) through the STK PamChips to promote interactions between lysates and immobilized peptides. After washing, detection of phosphorylation activity was performed with a mixture of a secondary fluorescein isothiocyanate (FITC)-labeled antibody. This mixture was pumped twice per cycle, with images being recorded every 5 cycles for 30 cycles.

Evaluation of array pictures was performed using the BioNavigator platform (V6.3; PamGene International BV) interfaced with R, assessing the intensity of the signals of phosphorylated peptides, which were quantified with the Evolve3 application from BioNavigator and log2-transformed. Basal STK signals per peptide for each sample were visualized in an auto-scaled heatmap. The nominal coefficient of variation (CV) for each peptide was calculated with the two-component error fit model using the overall mean intensity of signal, followed by evaluation of CVs between samples. The ComBat batch correction normalization [29], using empirical Bayes frameworks, was used to correct for batch effects associated with physical limitations of the methodology, which allows the analysis of four samples per chip, three chips at a time. Samples were also randomized when loaded to the PamChip to account for possible batch variations. To assess the differences between treatment conditions, analyses were performed by building the following contrasts (i.e., pairwise comparisons): “cAMP vs C,” “Agle 3h vs cAMP,” “Agle 6h vs cAMP,” “Mife 3h vs cAMP,” and “Mife 6h vs cAMP.” A list of significant differentially phosphorylated peptides (*P* < 0.05) for each contrast was acquired with a two-group unpaired *t*-test. The prediction of upstream STKs was finally performed with the Kinexus Kinase Predictor tool (Kinexus Bioinformatics Corporation, Vancouver, Canada). A cut-off for the final score of 1.2 was selected for the present analysis, and a full list of predicted STKs for all contrasts is presented in Supplemental Table 1. The representation of kinases with a predicted significantly different activity in each contrast was performed with upstream kinase score plots, which allow a hierarchical visualization of the most affected STKs, and with the online tool Coral (http://phanstiel-lab.med.unc.edu/CORAL/), using as input the median final score and median kinase statistic for each STK. Finally, functional analysis using KEGG pathways was obtained using the Proteomaps tool (https://proteomaps.net/, [30]). Overlap between kinases predicted to be affected under different treatment conditions was assessed with Venn diagrams using the online tool VENNY (https://bioinfogp.cnb.csic.es/tools/venny/; V2.1).

### RNA isolation, reverse transcription, and semi-quantitative real-time TaqMan PCR

In experiments evaluating effects of selected kinases, cells were washed twice with ice-cold PBS at the end of treatment and harvested with TRIzol for the isolation of total RNA (Invitrogen, Carlsbad, CA, USA). Total RNA isolation and subsequent semi-quantitative TaqMan PCR were conducted as previously described [31, 32] and according to the manufacturers’ protocols. The concentration and purity of total RNA were measured with a NanoDrop 2000 Spectrophotometer (Thermo Fisher Scientific AG). Subsequently, total RNA from each sample was treated with RQ1 RNase-free DNase (Promega, Duebendorf, Switzerland) to remove possible genomic DNA contamination. Complementary DNA (cDNA) was synthesized with the MultiScribe Reverse Transcriptase, using random hexamers as primers along with other RT reagents (Applied Biosystems by Thermo Fisher, Waltham, MA, USA). The reactions were carried out in an Eppendorf Mastercycler thermocycler (Vaudaux-Eppendorf AG, Basel, CH).

Semi-quantitative real-time TaqMan PCR was conducted in duplicates, using cDNA corresponding to 50 ng total RNA from each sample for each target gene, with the FastStart Universal Probe Master (Roche Diagnostics AG, Rotkreutz, Switzerland) in an automated ABI PRISM 7500 Sequence Detection System fluorometer (Applied Biosystems). For this, pre-designed and commercially available assays from Applied Biosystems were used. When unavailable, self-designed primers and 6-carboxyfluorescein (6-FAM) and 6-carbocytetramethylrhodamine (TAMRA) labeled probes were purchased from Microsynth (Balgach, Switzerland). The self-designed primers and probes had been constructed previously based on published coding sequences and underwent evaluation of probe efficiency to ensure it was approximately 100%, as previously described [31, 32]. Details regarding the primers and probes used can be found in Table 1. Negative controls for real-time PCR consisted of autoclaved water or non-reverse-transcribed RNA instead of cDNA (the RT-minus control). The ∆∆Ct method was used to determine relative gene expression. *KDM4A*, *PTK2*, and *EIF4H* were initially chosen as independent endogenous reference genes for quantification due to the reported stability of their expression in canine reproductive tissues [33]. However, following evaluation of their stability with the online tool RefFinder [34], *EIF4H* was excluded from the analysis. To address non-normal distribution of real-time PCR data, logarithmic transformation was employed, and the results are presented as geometric means (Xg) ± geometric standard deviation (SD).

**Table 1:** List of TaqMan systems used for the semi-quantitative real-time TaqMan PCR

Primer gene name	Accession numbers	Primer sequence		Product length (bp)
*PTGES*	NM_001122854	Forward Reverse TaqMan probe	5′-GTC CTG GCG CTG GTG AGT-3′ 5′-ATG ACA GCC ACC ACG TAC ATC-3′ 5′-TCC CAG CCT TCC TGC TCT GCA GC-3′	89
*PRLR*	HQ267784	Forward Reverse TaqMan probe	5′-GGA TCT TTG TGG CCG TTC TTT-3′ 5′-AAG GAT GCA GGT CAC CAT GCT AT-3′ 5′-ATT ATG GTC GTA GCA GTG GCT TTG AAA GGC-3′	92
*PGR*	NM_001003074	Forward Reverse TaqMan probe	5′-CGA GTC ATT ACC TCA GAA GAT TTG TTT-3′ 5′-CTT CCA TTG CCC TTT TAA AGA AGA-3 5′-AAG CAT CAG GCT GTC ATT ATG GTG TCC TAA CTT-3′	113
*PTGS2 (COX2)*	HQ110882	Forward Reverse TaqMan probe	5′-GGA GCA TAA CAG AGT GTG TGA TGT G-3′ 5′-AAG TAT TAG CCT GCT CGT CTG GAA T-3′ 5′-CGC TCA TCA TCC CAT TCT GGG TGC-3′	87
*IGF1*	NM_001313855	Applied Biosystems, prod nr. Cf02627846_m1		104
*KDM4A*	XM_005629106	Applied Biosystems, prod nr. Cf02708629_m1		96
*PTK2*	XM_005627993	Applied Biosystems, prod nr. Cf02684608_m1		104
*EIF4H*	XM_014114129	Applied Biosystems, prod nr. Cf02713640_m1		136

### Protein preparation and western blot analysis

For protein expression analysis, cells were washed and collected by scraping in ice-cold PBS at the end of the experiments and centrifuged at 1000 g for 10 min. The pellet was resuspended in Net-2 Lysis Buffer (50 mM Tris-HCl, pH 7.4, 300 mM NaCl, 0.05% NP-40) containing 10 μL/mL Protease Inhibitor Cocktail (Sigma-Aldrich Chemie GmbH). To perform nuclear disruption and ensure homogenization, samples were sonicated with a Vibra-Cell 75186 (Sonics & Materials, Inc., Newtown, CT, USA) at 75 W for two cycles of 10 s, on ice. Protein amounts were measured with the Bradford assay, and protein concentration was normalized with sample buffer (25 mM Tris-HCl, pH 6.8, 1% SDS, 5% beta-mercaptoethanol, 10% glycerol, 0.01% bromophenol blue). The expression of selected proteins was analyzed via SDS-PAGE western blot, following our previously published protocol [35] with slight modifications. A total of 20 μg of protein was loaded into 10% polyacrylamide gels and electrophoretically separated at 120 V. The separated proteins were then transferred onto methanol-activated polyvinylidene difluoride membranes in a wet tank at 100 V for 1 h. Membranes were blocked with 5% low-fat milk in PBS/0.25% Tween 20 (PBST) for 1 h at ambient temperature, followed by overnight incubation with primary antibodies diluted in 2.5% low-fat milk at 4 °C. A list of all antibodies used in the western blot analysis, including dilutions, is presented in Table 2. On the following day, after washing with PBST, horseradish peroxidase (HRP)-conjugated secondary antibodies, targeting the species where primary antibodies were developed, were applied at 1:7500 dilution to the membranes for 1 h at ambient temperature. Signals were detected with the SuperSignal West Chemiluminescent Kit substrate (Thermo Fisher Scientific AG) visualized in a Chemi-Doc XRS+ System and Image Lab Software (both from Bio-Rad Laboratories). Semi-quantification of protein expression was conducted using the ImageJ software (US National Institutes of Health, Bethesda, Maryland, USA) by normalizing the standardized optical density (SOD) of the target protein against BACTIN on re-blotted membranes. For this, antibodies were stripped with 0.1 M glycine (pH = 2.6) for 1 h at ambient temperature and each membrane was re-probed with the anti-BACTIN antibody. Numerical data for semi-quantification of proteins are presented as mean ± SD.

**Table 2:** List of antibodies used for western blot analysis

Antibody	Company	Reference number	Host	Dilution
P-cJUN	Cell Signaling Technology	Ser73	Polyclonal rabbit	1:500
cJUN	Cell Signaling Technology	60A8	Polyclonal rabbit	1:1000
P-CREB	Cell Signaling Technology	87G3	Monoclonal rabbit	1:1000
CREB	Cell Signaling Technology	48H2	Monoclonal rabbit	1:1000
P-p44/42 MAPK (Erk1/2)	Cell Signaling Technology	9101	Polyclonal rabbit	1:1000
p44/42 MAPK (Erk1/2)	Cell Signaling Technology	9102	Polyclonal rabbit	1:1000
BACTIN	Santa Cruz Biotechnology	sc-69879	Monoclonal mouse	1:1000
Goat anti-mouse HRP-labeled secondary IgG	Promega	W402B	Goat anti-mouse IgG	1:7500
Goat anti-rabbit HRP-labeled secondary IgG	Thermo Fisher Scientific	31460	Goat anti-rabbit IgG	1:7500

### Statistical analysis

Statistical analyses of mRNA and protein relative expression were performed using GraphPad 3.06 software (GraphPad Software, San Diego, CA, USA). For this, one-way ANOVA was conducted followed by a Tukey–Kramer multiple-comparisons post hoc test when *P* was below the 0.05 threshold.

## Results

### Kinomics analysis

#### Initial assessment and quality control

**Figure 1:**
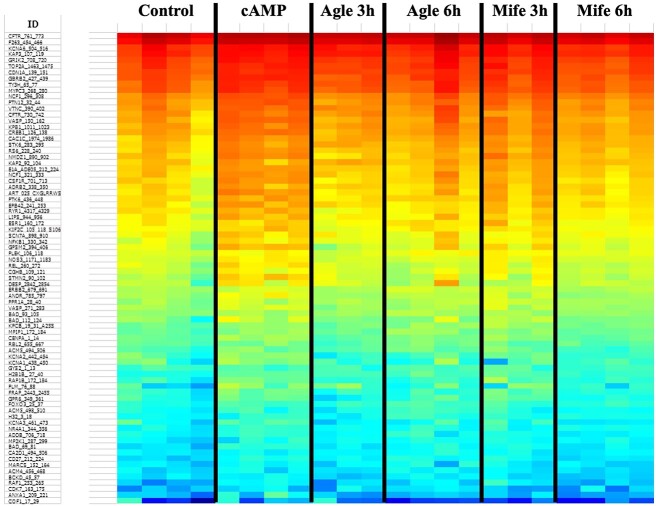
Heatmap presenting the predicted activation of STKs based on the phosphorylation levels of their substrates modulated in DUS cells under different treatment conditions. Signals for each sample are presented in an auto-scaled heatmap comparing phosphorylation levels between all detected STK substrates. Colors ranging from red to blue indicate a gradient of high to low phosphorylation levels, respectively. An apparently increased phosphorylation level can be observed in the cAMP group (decidualized DUS cells). Some intra-group variation can be observed, mainly in antigestagen-treated groups (Agle = aglepristone, Mife = mifepristone).

In the initial assessment of the kinomics results, peptides with low intensity or showing a CV >50% were removed from analysis. While the quality control criteria for signal strength and number of peptides detected (> 90 peptides) were met, the coefficient of variation between replicates was between 20 and 30%. For this reason, following the visual analysis of the heatmap, one sample from the aglepristone 3 h group and one from the mifepristone 3 h group were considered to be outliers and removed from further downstream analysis. Following the batch correction normalization using ComBat, some variations between the samples in each group could still be observed (Figure 1), possibly associated with intra- and/or inter-assay variability. Nevertheless, a qualitative visualization of the heatmap suggested an enrichment of peptides phosphorylated in the cAMP (decidualized) group, when compared with control or antigestagen-treated groups (Figure 1).

#### Decidualization-associated effects

**Figure 2:**
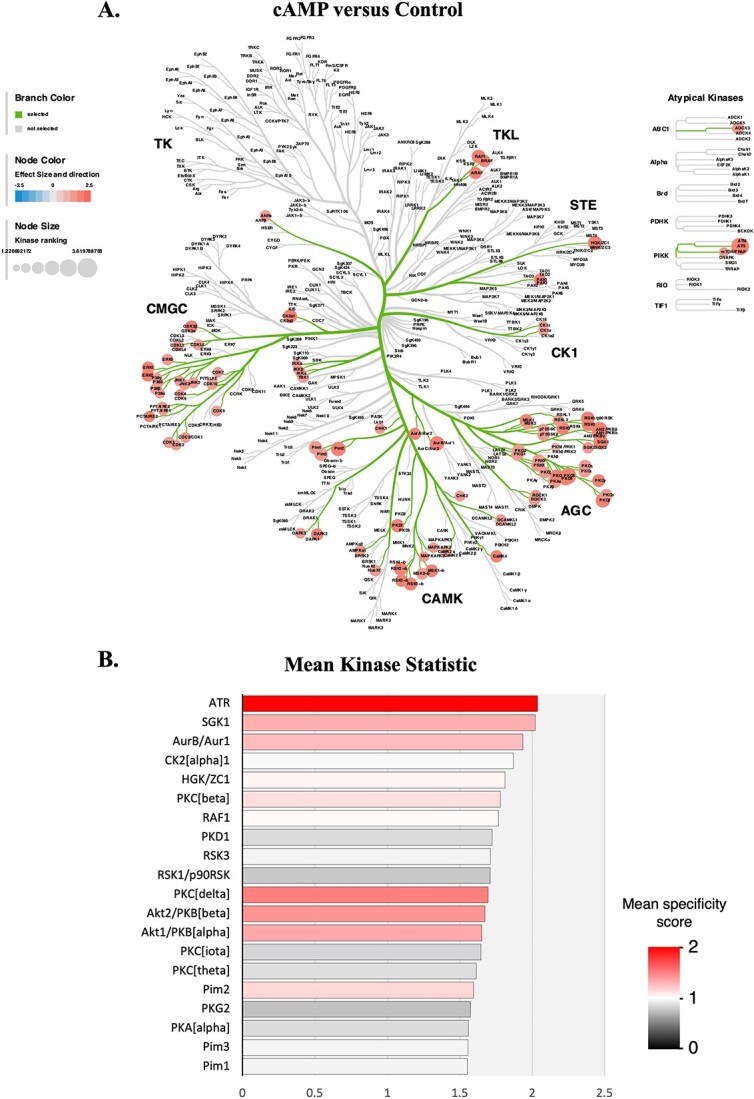
Visualization of STKs with predicted increased activity in the contrast “cAMP (decidualized) over Control (non-decidualized).” The activity of 85 STKs was predicted to be increased in DUS cells after decidualization (*P* < 0.05). (**A**) Representation of affected kinases in a Coral plot, identifying most of the kinases affected as belonging to the kinase families AGC, CAMK, and CMGC. (**B**) Upstream kinase score plot showing the 20 kinases with the highest mean specificity score listed, ranked by mean kinase statistic, for the same contrast.

To evaluate changes in the DUS cells kinome after decidualization, a pairwise comparison of DUS cells from cAMP-treated and control groups was performed, and activated STKs were predicted using the Kinexus Kinase Predictor tool. In total, 85 STKs were determined to have significantly increased activity after the decidualization of DUS cells, with no kinases being predicted to have decreased activity (Figure 2, Supplementary Table 1, Supplementary Figure 1A). Among them were (Figure 2, Supplementary Table 1) members from the AGC family including PKA, PKC, PKG, and Akt (Figure 2B); CMCG family members, including several MAPKs such as ERK1/-2 and p38 MAPKs, and IkappaB kinases (IKKs); members of the CAMK family including different ribosomal s6 kinases (RSKs) and calcium/calmodulin dependent protein kinase IV (CaMK4); and members of the tyrosine-kinase like kinase (TKL), STE, and phosphatidylinositol 3-kinase-related kinases (PIKK) families, with ataxia telangiectasia and Rad3-related protein (ATR) being predicted to be the more strongly activated kinase in response to decidualization. A functional analysis of the kinases modulated by decidualization was performed with proteomaps, focusing on protein function and using the KEGG pathway classification. Among the main functional terms enriched by cAMP in DUS cells were those associated with MAPK, forkhead box protein O (FOXO), and nuclear factor kappa-light-chain-enhancer of activated B cells (NFκB) signaling pathways, but also with the chemokine signaling pathway, gap junctions, and the cell cycle (Supplementary Figure 1B).

#### Antigestagen-mediated effects

**Figure 3:**
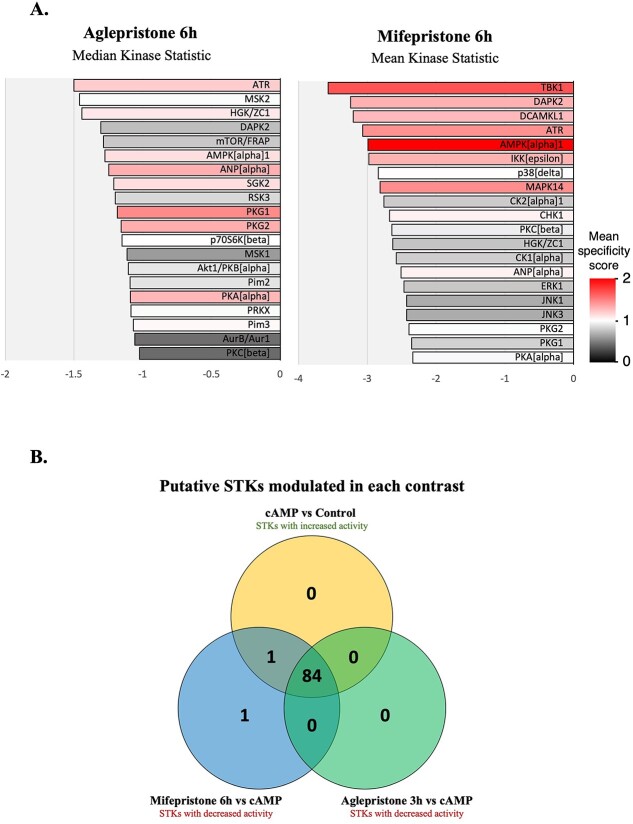
(**A**) Upstream kinase score plot showing the 20 kinases with the highest mean specificity score in decidualized DUS cells treated with aglepristone or mifepristone for 6 h. (**B**) Venn diagram showing the overlap of the predicted STKs presenting an increased activity in the contrast “cAMP (decidualized) versus Control (non-decidualized)” with those presenting a decreased activity in the contrasts “Aglepristone 3h versus cAMP” and “Mifepristone 6h versus cAMP” (time points showing the strongest effects for each of the antigestagens).

The effects induced by PGR blocking were assessed by comparing the different antigestagen-treated groups with the cAMP (decidualized) group. Antigestagen treatment of decidualized DUS cells was associated with a predicted decrease in the activity of all significantly modulated kinases (Figure 3A, Supplementary Table 1, Supplementary Figures 2–4). The treatment of decidualized DUS cells with aglepristone for 3 h was associated with the modulation of 84 STKs, whereas 59 kinases were predicted to be significantly modulated after 6 h (Supplementary Table 1, Supplementary Figures 2A–B and 3). As for mifepristone, while only 39 STKs could be identified from the peptides phosphorylated by lysates of cells treated for 3 h, a total of 86 STKs were predicted to have decreased activity after 6 h of treatment (Supplementary Table 1, Supplementary Figures 2C–D and 4). The kinases predicted to be deactivated under all antigestagen-treatment conditions included the AGC family members PKA, PKC, and PKG, in addition to MAPK activated protein kinase (MAPKAK) -2 and -3, and the CMGC member cyclin dependent kinase 10 (CDK10) (Supplementary Table 1, Supplementary Figures 2–4). Among the kinases most affected after 6 h of treatment with both antigestagens were ATR, as well as AMP-activated protein kinase (AMPK), PKA, and PKGs (Figure 3A, Supplementary Table 1, Supplementary Figure 2). In addition, the activity of ERK1 and 2 was predicted to be decreased by aglepristone after 3 h, and by mifepristone after 3 (for ERK1) and 6 h (both kinases) (Supplementary Table 1, Supplementary Figures 2–4). At the functional level, the kinases modulated by antigestagens at all time points enriched functional terms associated with the predicted affected activity of MAPK, FOXO, and NFκB signaling pathways, but also with gap junctions and the cell cycle (Supplementary Figure 5).

When overlapping the kinases predicted to be significantly modulated in different contrasts, virtually all of the STKs with an increased activity in the contrast “cAMP vs Control” showed a decreased activity in both “Agle 3h vs cAMP” and “Mife 6h vs cAMP” (Figure 3B). The only exceptions were the kinase protein kinase N1 (PKN1/PRK1), which was not identified in the contrast “Agle 3 h vs cAMP,” and ribosomal S6 kinase-like protein 1 (RSLK1), which was only modulated after 6 h of treatment with mifepristone (Figure 3B).

### Modulation of the activity of selected kinases in decidualized DUS cells

Following the observations from the kinomics analysis, the involvement of selected STKs in the physiology of DUS cells was further evaluated. DUS cells decidualized with cAMP were treated with specific inhibitors targeting PKA (H89), PKC (GF-109203X/GFX), and the ERK1/2 activators MEK1/2 (U0126). Treatments with mifepristone were also performed for comparative purposes.

#### Inhibitors of PKA and MEKs evoke stronger effects than those of PKC upon the expression of decidualization markers in decidualized DUS cells

**Figure 4:**
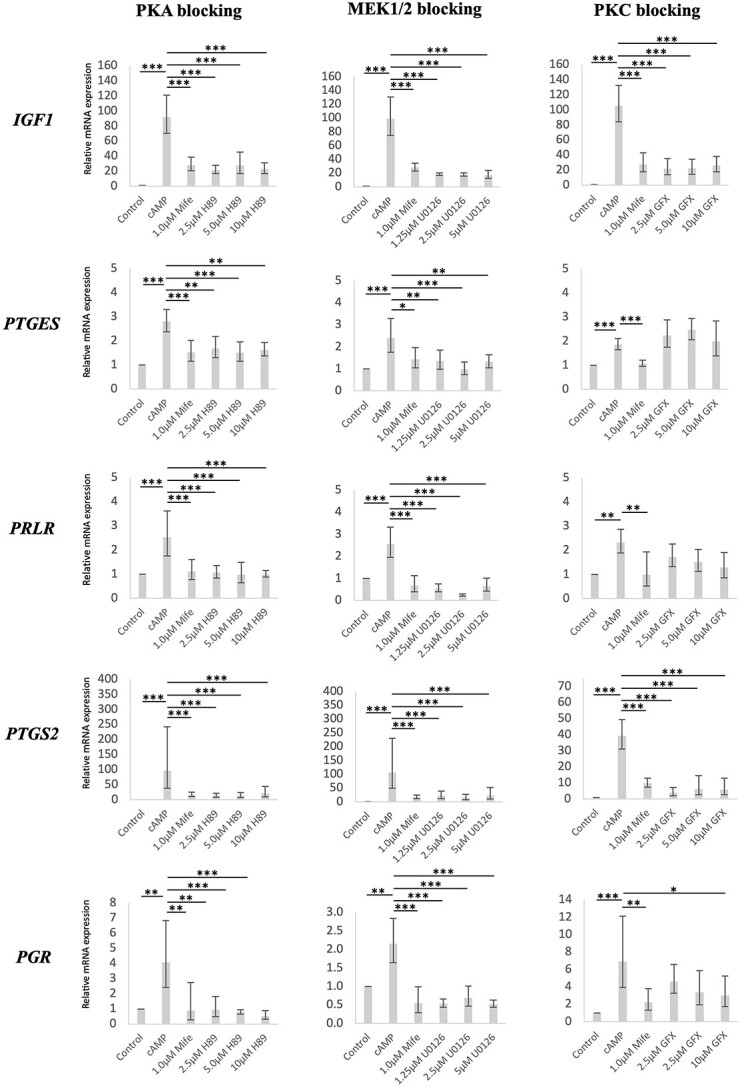
Modulatory effects of inhibitors of different STKs on decidualization markers in decidualized DUS cells. Following decidualization (0.5 mM cAMP, 72 h), DUS cells were treated for 6 h with mifepristone or inhibitors of PKA (H89), PKC (GFX), or MEK1/2 (U0126) activity, respectively. The relative gene expression of insulin-like growth factor (*IGF*)-1, prostaglandin E2 synthase (*PTGES*), prolactin receptor (*PRLR*), prostaglandin-endoperoxidase synthase 2 (*PTGS2/COX2*), and progesterone receptor (*PGR*) was evaluated by semi-quantitative real-time (TaqMan) PCR. Numerical data are presented as geometric mean ± geometric SD. Effects of treatment were evaluated with one-way ANOVA, revealing *P* <0.0001 for all treatments and factors, except for *PRLR* after treatment with GFX (*P* = 0.0021). When *P* <0.05, analysis was followed by a Tukey–Kramer multiple-comparisons post hoc test. Bars differ at ^*^P < 0.05, ^*^^*^P < 0.01, ^*^^*^^*^P < 0.001. Only the statistical results with relation to the cAMP group are presented in the figure.

The initial assessment of STK-associated effects on decidualized DUS cells was performed by focusing on the transcriptional availability of the well-established decidualization markers *IGF1*, *PTGES*, *PRLR*, *PTGS2*, and *PGR* [16, 18, 19]. Serving as the positive control, the transcriptional availability of all of these decidualization markers was increased after cAMP-induced decidualization (*P* < 0.01, Figure 4). Furthermore, the inhibition of PGR with mifepristone was associated with decreased mRNA amounts of all investigated factors in decidualized DUS cells (*P* < 0.01, Figure 4). Treatment with the inhibitors of both PKA (H89) and MEK1/2 (U0126) at all dosages led to a significant reduction in the number of transcripts encoding for *IGF1*, *PTGES*, *PRLR*, *PTGS2*, and *PGR* when compared to non-treated decidualized cells (*P* < 0.01, Figure 4). Similarly, the usage of a PKC inhibitor (GF-109203X/GFX) at all tested concentrations was associated with the downregulation of *IGF1* and *PTGS2* (*P* < 0.001, Figure 4), and of *PGR* at a concentration of 10 μM (*P* < 0.05, Figure 4). However, the mRNA amounts of *PRLR* and *PTGES* remained unaffected by GF-109203X (*P* > 0.05, Figure 4).

The MAPK inhibitor U0126 specifically targets the MAP2K kinases MEK1/2, with ERK1/2 being their only known substrates [36]. Thus, to assess if the effects of U0126 on the transcriptional availability of decidualization markers in DUS cells were mediated through ERK1/2, changes in the protein amounts of phosphorylated and total p44 (ERK1) and p42 (ERK2) were assessed. Surprisingly, treatment of decidualized DUS cells with U0126 for 6 h did not affect the protein expression profile of p44/42 MAPKs (*P* > 0.05, Supplementary Figure 6).

#### PKA and PKC signaling induce distinct phosphorylation patterns of cJUN and CREB in decidualized DUS cells

To further confirm the functional involvement of selected kinases and to explore intracellular signaling pathways in decidualized DUS cells, the availability of well-established cAMP responders cJUN and CREB (cAMP response element-binding protein), as well as their transcriptionally active phosphorylated (P) forms, was investigated. The effects of decidualization, the inhibition of PGR, and suppression of PKA and PKC activity were assessed.

**Figure 5:**
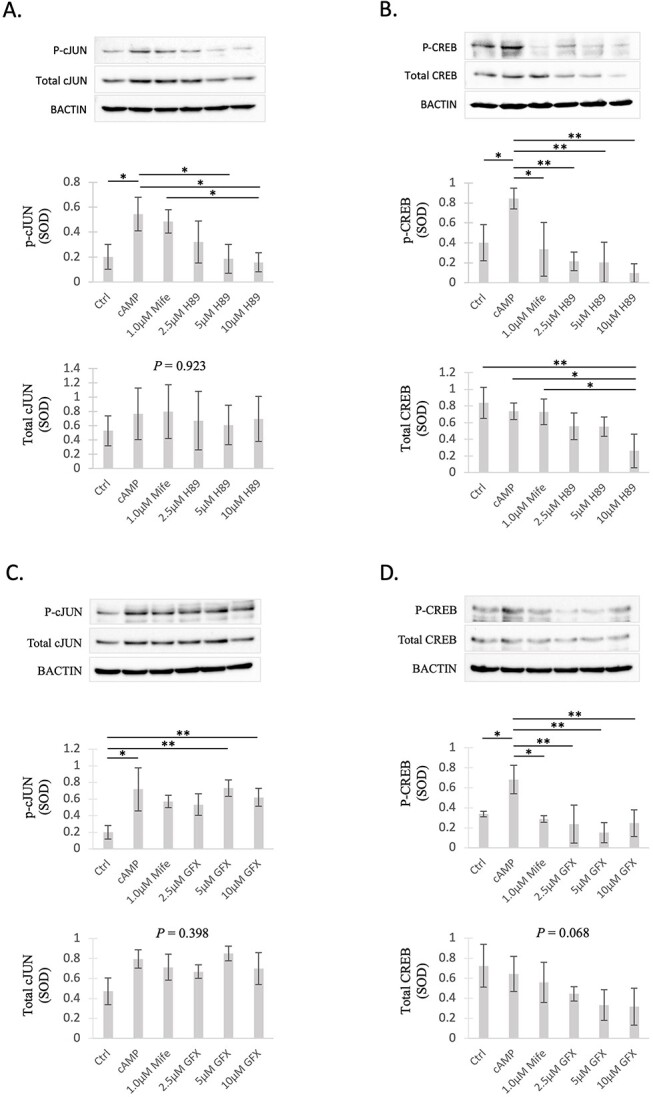
Protein expression of the transcription factors cJUN and CREB in DUS cells in response to the inhibition of PGR (mifepristone), PKA (H89), or PKC (GFX). Representative immunoblots are presented for each analyzed factor under each treatment condition. Standardized optical density (SOD) of (**A**, **C**) phosphorylated (P) and total cJUN (**B**, **D**), and CREB/P-CREB (both ~43 kDa) were normalized against BACTIN (45 kDa). Effects of treatment were evaluated with one-way ANOVA, revealing (**A**) *P* = 0.0061 for P-cJUN and 0.923 for total cJUN; (**B**) *P* = 0.0016 for P-CREB and 0.0095 for total CREB; (**C**) *P* = 0.006 for P-cJUN and *P* = 0.398 for total cJUN; (**D**) *P* = 0.0024 for P-CREB and 0.068 for total CREB. When *P* <0.05, analysis was followed by a Tukey–Kramer multiple-comparisons post hoc test. Numerical data are presented as mean ± SD. Bars differ at ^*^P < 0.05, ^*^^*^P < 0.01.

Following cAMP-induced decidualization, a significant increase in the protein expression of P-cJUN and P-CREB (*P* < 0.05) was observed in all experiments, whereas the total amounts of both transcription factors were unaffected (*P* > 0.05) (Figure 5A–D). Treatment with mifepristone (1 μM over 6 h) resulted in a significant decrease in the expression of P-CREB in decidualized DUS cells (*P* < 0.05, Figure 5B and D). However, no significant changes in the protein amounts of total CREB (*P* > 0.05, Figure 5B and D) and total cJUN or P-cJUN (*P* > 0.05; Figure 5A and C) were identified in response to the antigestagen.

With regard to the STKs, inhibition of PKA with 5 and 10 μM H89 resulted in a significant decrease in P-cJUN availability (*P* < 0.05; Figure 5A). Furthermore, all tested concentrations of H89 resulted in lower amounts of P-CREB in decidualized DUS cells (*P* < 0.01, Figure 5B). As for PKC inhibition, no significant differences were observed regarding the amounts of P-cJUN in DUS cells when compared to non-treated decidualized cells (*P* > 0.05, Figure 5C). However, GFX had suppressive effects on the protein amount of P-CREB in decidualized stromal cells (*P* < 0.01, Figure 5D). Finally, the total content of cJUN protein remained stable across all treatment groups (*P* > 0.05, Figure 5A and C). In contrast, inhibition of PKA with 10 μM of H89 (Figure 5B), but not of PKC (*P* > 0.05, Figure 5D), led to a significant decrease in the total protein expression of CREB when compared with non-decidualized and decidualized DUS cells (*P* < 0.05).

### Transcriptomics effects upon kinases

**Figure 6:**
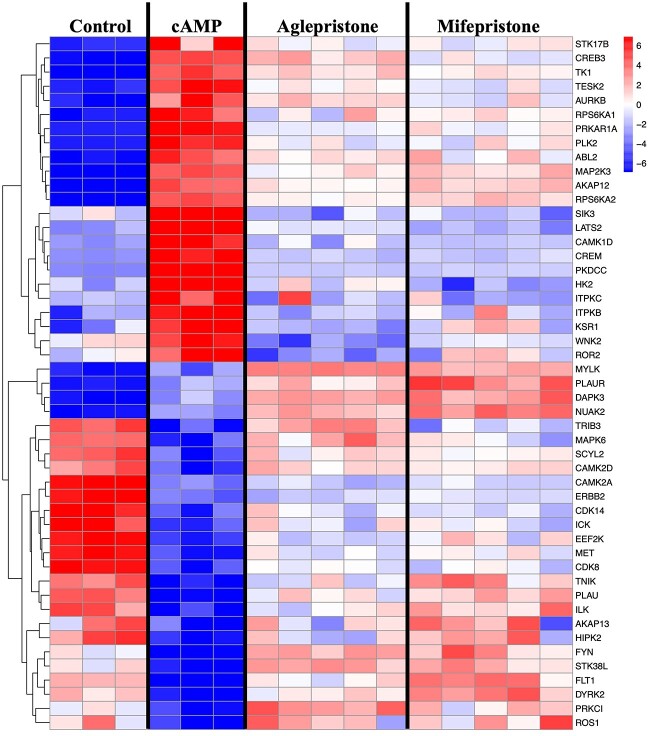
Transcriptional modulation of 49 differently expressed genes (DEGs, *P*, and FDR < 0.01) encoding for kinases and associated factors in all contrasts (“cAMP vs control,” “Aglepristone vs cAMP,” “Mifepristone vs cAMP”). The previously published dataset GSE213788 available in NCBI’s Gene Expression Omnibus was used for this analysis [21].

Having observed the effects evoked by antigestagens upon kinase activity, as well as the effects of mifepristone upon P-CREB, and taking into account that PGR is a nuclear transcriptional factor, we hypothesized that antigestagen-mediated effects on the activity of the predicted kinases could be, at least in part, associated with the modulation of their transcriptional availability. Thus, to explore some of these possible effects, we referred to the previously published dataset GSE213788 available through NCBI’s Gene Expression Omnibus [21]. This dataset was generated to assess changes in the transcriptional signature of DUS cells after decidualization, identifying 3316 differently expressed genes (DEGs, *P* < 0.01 and FDR < 0.01) [21]. In the same study, response to mifepristone and aglepristone treatment revealed 1558 and 1400 DEGs, respectively [21]. By utilizing this dataset, we identified 49 DEGs associated with the term “kinases” (Figure 6). Of these, 23 kinases and associated factors, which were significantly upregulated following cAMP-induced decidualization, had a negative transcriptional regulation after treatment with antigestagens (Figure 6). These included A-kinase anchoring protein 12 (*AKAP12*), *CAMK1D*, and *CREB3*, along with protein kinase cAMP-dependent type I regulatory subunit alpha (*PRKAR1A*), ribosomal protein S6 kinase A1 (*RSPS6KA1*), and *RSPS6KA2*. Following an opposite pattern of expression, 26 kinase-associated DEGs that were significantly downregulated during the cAMP-induced decidualization had increased transcriptional amounts in response to type II antigestagens, including death associated protein kinase 3 (*DAPK3*), *CAMK2A* and *-D*, *CDK8* and *-14*, integrin-linked kinase (*ILK*) and *AKAP13*, as well as the tyrosine kinases *MET* and Fms Related Receptor Tyrosine Kinase 1 (*FLT1*).

## Discussion

Here, the kinomics approach was applied for the first time to investigate the function of decidualized stromal cells. Intra- and inter-group variations in the kinomics profiles might be further associated with technical limitations of the methodology. The length of treatment used was chosen based on studies investigating gene and/or protein expression in DUS cells [19]. Consequently, since kinase activity fluctuates over time [37], the present analysis highlights the activity of STKs at a specific time point. It is, thus, possible that some changes in the kinomics pattern could not be detected. The PamChip technology was initially designed to evaluate human and murine kinases. To the best of the authors’ knowledge, this study represents its first application in the canine species. Therefore, the presence of species-specific differences could contribute to the observed variation within groups. Moreover, each PamChip can be loaded with only four samples, with a maximum of three chips being run in the PamStation for each analysis. To account for these possible batch effects, samples from each group were randomly allocated to different runs and chips, and extra steps in the statistical analysis were taken to normalize the results obtained. Samples identified as outliers were removed from the downstream evaluation of results. Despite all these limitations, we found clear effects of decidualization and antigestagens on kinetic activity in DUS cells, revealing new clues regarding the involvement of a wide range of STKs in the decidualization process, utilizing the dog as a model.

Canine decidual cells are a significant focus of research in studies related to canine reproduction because of their expression of nuclear PGR and the potential for antigestagen-mediated functional modifications. Based on the inducibility of decidualization by cAMP, the activation of PKA and other ACG kinases in decidualized canine stromal cells was expected. Yet, together with PKA, more than 80 other STKs were predicted to be activated. Among the functional terms enriched for these STKs were the FOXO signaling pathway, gap junctions, and cell cycle regulation. These terms fit with decidualization being characterized by mesenchymal–epithelial transition of DUS cells, associated with increased secretory activity, increased cell–cell adhesion, and modulation of extracellular matrix components [18–21]. Moreover, in a prior transcriptional study, FOXO signaling, linked in the present analysis to STKs such as Akt, was suggested to play a role in canine decidualization [21]. Likewise, in human endometrial stromal cells, FOXO1, as a representative of this family, is one of the earliest transcription factors responding to a decidualization stimulus [38, 39]. FOXO1 has also been implicated in mediating apoptosis of decidualized human stromal cells following the withdrawal of P4 [40].

The enrichment of NFκB signaling for the kinases modulated after decidualization was accompanied by the activation of IKKs, which are mediators of cellular response to inflammation through the activation of NFκB [41, 42]. In humans, uterine levels of NFκB are increased during the premenstrual phase or in the decidua during the first trimester, and the in vitro decidualization of stromal cells is associated with enrichment of this signaling pathway [43, 44]. In DUS cells, NFκB was identified among upstream regulators associated with decidualization, with regulators like NFκB inhibitor alpha (NFκBIA) being modulated after decidualization [21]. Thus, the increased activity of IKKs identified in the kinomics analysis appears to support the enrichment of NFκB signaling after decidualization in DUS cells. Although still requiring confirmation, this association may also relate to the previously suggested immunoreactive functions of canine decidual cells [21].

The increased activation of several members of the CAMK family, including the activation of CAMK1D that was also reported to be upregulated in the transcriptomic data, further attracted our attention. Activation of CAMKs occurs in response to elevated intracellular calcium (Ca^2+^) concentrations along with the presence of calmodulin [45]. Increased Ca^2+^ acts as an important messenger during decidualization of human endometrial stromal cells [46], and its fluctuations alters intracellular cAMP levels, thereby affecting cellular differentiation [47]. Thus, the involvement of Ca^2+^ signaling in the regulation of canine decidualization appears plausible. Moreover, various cyclin-dependent kinases (CDKs) belonging to the CMGC family appear to be involved in our cAMP-induced decidualization approach. CDKs hold significant importance in regulating the cell cycle, cell proliferation, and cell migration [48–50], functions that are also relevant during embryo implantation and canine decidualization [10, 19, 21]. In fact, CDKs were reported to be involved in the regulation of the cell cycle and initiation of cell differentiation during human decidualization both in vitro and in vivo [51]. However, targeted studies in the canine species are still required to confirm these mechanisms.

The negative effects of antigestagens upon decidualization are well documented, both for the dog [19, 21] and for other species, e.g., humans and rodents [52–54]. Remarkably, treatment with type II antigestagens negatively influenced the activity of virtually all kinases activated after the cAMP-induced decidualization, indicating the functional kinetic reversal of the process and affecting virtually all STKs induced by decidualization. The effects on the kinases varied between the two antigestagens with the length of treatment. Since aglepristone is a chemical derivate of mifepristone, both type II antigestagens have similarities in structure and mechanisms of action, with a higher affinity to the PGR than P4 itself and acting as transdominant repressors (reviewed in [13]). Despite these similarities, mifepristone and aglepristone have been shown to have some differences in their effects on the transcriptome of decidualized DUS cells [19, 21]. The temporal differences observed in their effects may indicate additional functional differences between both compounds.

While the kinomics approach provided new clues regarding a wide number of kinases affected by decidualization and PGR inhibition, it did not address the functional relevance of these factors. Hence, to provide initial insights into the potential functionality of some of the identified kinases, we inhibited selected kinases in decidualized DUS cells. We initially focused on PKA due to its importance as a mediator of cAMP signaling. PKC was selected for targeted studies as different isoforms of this STK were among the kinases predicted to be the most affected under different treatment conditions. Notably, the inhibition of PKA resulted in decreased gene expression of all the decidualization markers investigated, while no significant effects were observed on the transcriptional levels of *PTGES* or *PRLR* after PKC inhibition. The stronger effects of PKA, when compared with PKC, were also observed when assessing the activation of the transcription factors cJUN and CREB. The phosphorylation of CREB, increased after the cAMP-induced decidualization of DUS cells, was affected by the inhibition of both PKA and PKC. Interestingly, although the inhibition of PKA decreased the activation of cJUN, PKC inhibition did not alter protein levels of activated (i.e., phosphorylated/P) cJUN in decidualized cells. These findings indicate that both cJUN and CREB are downstream targets of cAMP through PKA in decidualized DUS cells, but only CREB appears to be regulated by PKC signaling. These results emphasize the importance of the cAMP/PKA pathway, mediated through cJUN, but also through CREB, in regulating decidualized cell function in the dog.

Another functional term enriched for the STKs activated in decidualized DUS cells was related to the MAPK signaling pathway. This was associated with the modulation of, i.a., ERK1/2 and p38 MAPKs. The activation of ERK1/2, and increased expression of their target genes, were observed in mice and humans at implantation sites, with the inhibition of ERK1/2 impairing the expression of decidualization markers [55]. On the other hand, both ERK1/2 and p38 MAP kinases were also associated with IL1β-induced disruptive mechanisms related to connexin-dependent cell-to-cell communication in decidualized human stromal cells [56]. Here, decidualized DUS cells were treated with U0126 to assess the importance of MAPKs in the expression of decidualization markers and, similar to the experiments targeting PKA activity, resulted in the decreased expression of all target genes. U0126 is an inhibitor of MEK1/2, which are assumed to be responsible for the activation of ERK1/2, their only known targets to date [36]. Therefore, it was surprising to observe that the activation levels of ERK1/2 remained unaffected after 6 h of treatment. Some off-target effects of U0126 have been reported [57–59], although the underlying mechanisms are not yet fully understood. Accordingly, it remains unclear whether any of these effects could potentially affect our results or if other, yet undiscovered, targets related to MAPK activities of U0126 could be involved. Moreover, as already mentioned, the present analysis did not account for time-dependent variations in kinase activities in response to stimuli. It is still plausible that the activation of ERK1/2 could undergo different time-dependent modulation to that experienced during the 6-h treatment. Nevertheless, in view of the notable detrimental impact on decidualization markers in DUS cells treated with the MAPK inhibitor, their involvement seems to be unquestionable, even though the exact signaling pathways affected by U0126 need further clarification.

Surprisingly, the inhibition of PGR with mifepristone resulted in decreased amounts of P-CREB without, however, affecting its total protein expression, and without any effects on the availability of total and P-cJUN. These cJUN-related findings were interesting because the kinomics analysis showed decreased activity of PKA, MAPKs, and other kinases that could potentially interact with the AP1 family of transcription factors, to which cJUN belongs. This also implies possible differences underlying the tissue and/or cell-specific downstream signaling pathways.

On the other hand, having seen the decreased kinomic activity, as well as decreased expression of P-CREB in antigestagen-treated cells, we hypothesized that the activity of kinases involved in the activation (i.e., phosphorylation) of transcriptional factors in decidualized cells could be regulated at the level of the availability of their transcripts, adding a new perspective to the PGR-mediated effects. To address this hypothesis, our previously published dataset derived from transcriptional analysis of changes associated with decidualization, as well as with antigestagen treatment of decidualized DUS cells, was utilized [21]. Among the 46 identified factors, we found the PKA subunit *PRKAR1A* [60] and the scaffolding protein and important regulator of PKA activity *AKAP12* [61], which were suppressed in decidualized DUS cells. The inclusion of these factors among the genes suppressed by antigestagens could account, at least in part, for the decreased activity of PKA signaling observed in the kinomics analysis, thus supporting our hypothesis. Furthermore, the decreased transcriptional availability of modulators of CREB activity, such as *RSPS6KA1* and *RSPS6KA2* [62], may be associated with the mifepristone-mediated reduction in P-CREB abundance. The same accounts for CAMP responsive element modulator (CREM), a transcriptional factor and another modulator of cAMP/PKA responses. The transcriptional availability of STKs like *CAMK1D*, *CDK8*, or *MAP2K3*, but also of genes encoding for the tyrosine kinases MET and vascular endothelial growth factor receptor 1 (VEGFR1, encoded by *FLT1*), was also suppressed in antigestagen-treated DUS cells*.* Thus, the transcriptional modulation of these kinases and their associated factors might reduce their functional availability, resulting in decreased kinase activity detected in response to aglepristone and mifepristone.

The possible PGR-mediated transcriptional regulation of kinase activity is one of the major functional findings from the present study. It is of translational potential, as well as of clinical importance, widening our view on the currently known PGR-mediated effects.

## Conclusion

This kinomics analysis is the first of its kind in studies addressing decidualization and pioneers the use of this methodology in the canine species. Several members of the AGC family (including PKA, PKC, and PKG kinases), but also representatives from the CAMK and CMGC families (such as CDK, glycogen synthase kinase (GSK), and various MAPKs), exhibited modulation in response to decidualization and after antigestagen treatment. Many of these factors, previously unexplored in decidual cells, have potential as novel intracellular targets for future investigations. In targeted studies, the effects of selective inhibition of PKA, PKC, and MEK1/2 revealed their involvement in regulating decidual cell physiology. The downstream signaling of PKA in DUS cells through the transcriptional factors cJUN and CREB was validated, further reinforcing the importance of cAMP/PKA pathway signaling in decidualized cells. The differences observed between PKC and PKA in DUS cells indicate variations in their downstream effects, reflected in different modulation of the expression of decidualization markers. The antigestagen-mediated deactivation of virtually all kinases activated by cAMP further highlights the importance of PGR signaling for the maintenance of decidual cell activity in dogs, in accordance with previous studies [19, 21]. The comparative analysis between the current kinomics and previous transcriptomics data [21] leads us to assume that the activity of kinases is at least partially regulated at the transcriptional level in a PGR-dependent manner, highlighting the translational significance of our approach.

## Supplementary Material

Suppl_Fig_1_ioad170

Suppl_Fig_2_ioad170

Suppl_Fig_3_ioad170

Suppl_Fig_4_ioad170

Suppl_Fig_5_ioad170

Suppl_Fig_6_ioad170

Supplemental_Table_1_ioad170

Supplemental_Figure_legend_ioad170

## Data Availability

Additional data underlying this paper will be shared on reasonable request to the corresponding author.
