# Supplementary figures and images for: Applying a novel kinomics approach to study decidualization and the effects of antigestagens using a canine model
Source: Biol Reprod. 2023 Dec 11;110(3):583–98. doi: 10.1093/biolre/ioad170 (PMC10941090; doi:10.1093/biolre/ioad170)

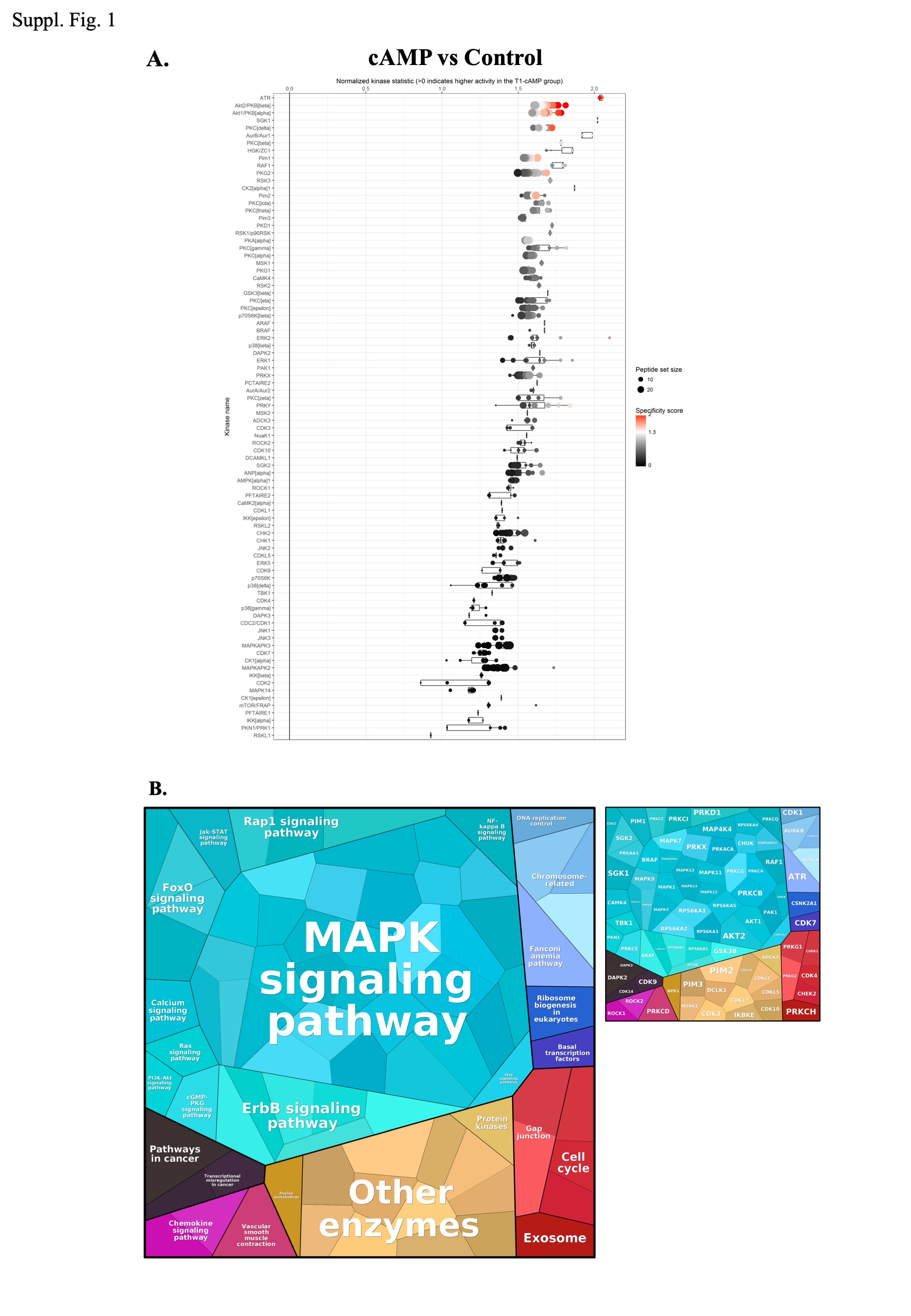

Supplement: Suppl_Fig_1_ioad170 [file suppl_fig_1_ioad170.jpeg]

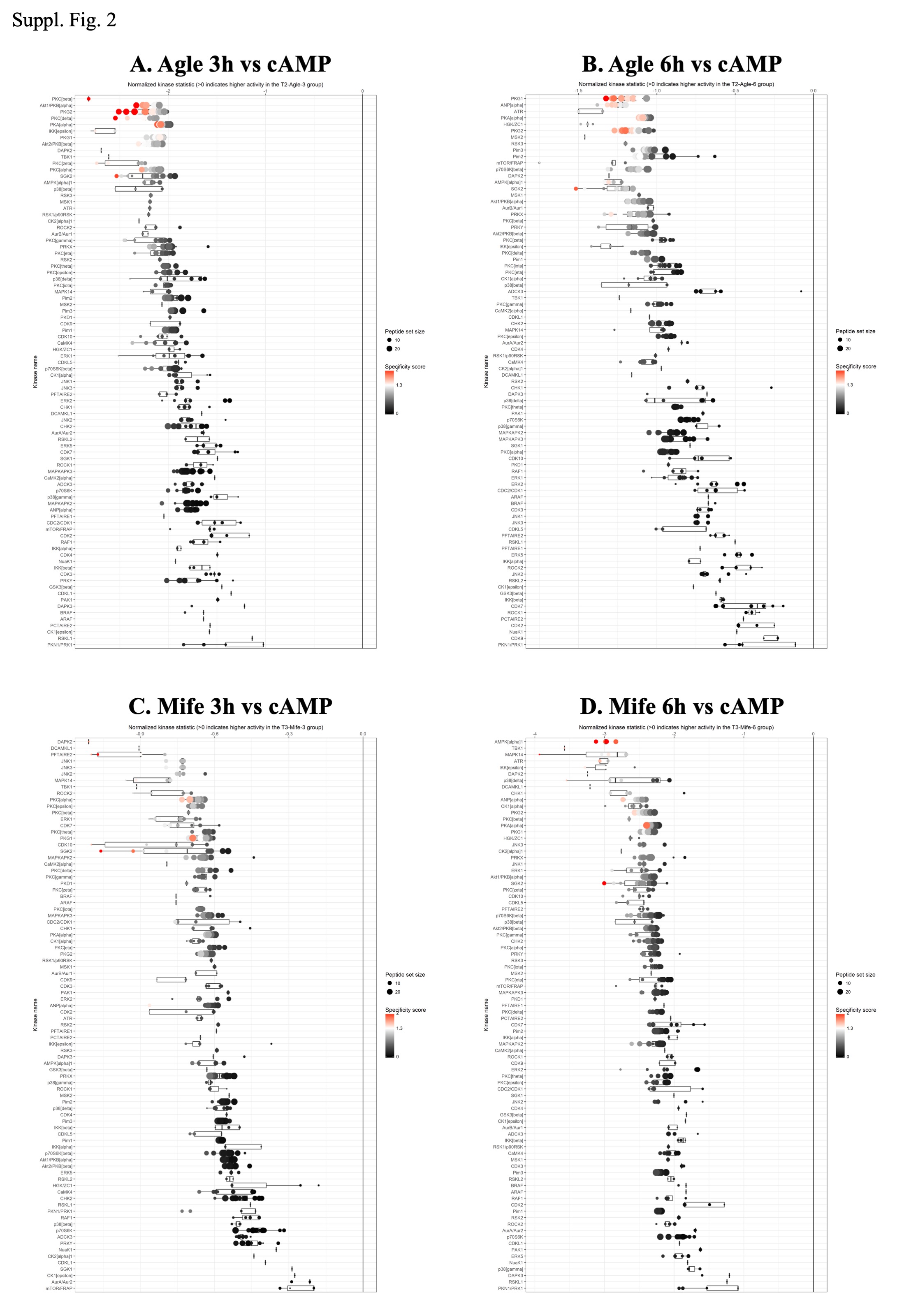

Supplement: Suppl_Fig_2_ioad170 [file suppl_fig_2_ioad170.jpeg]

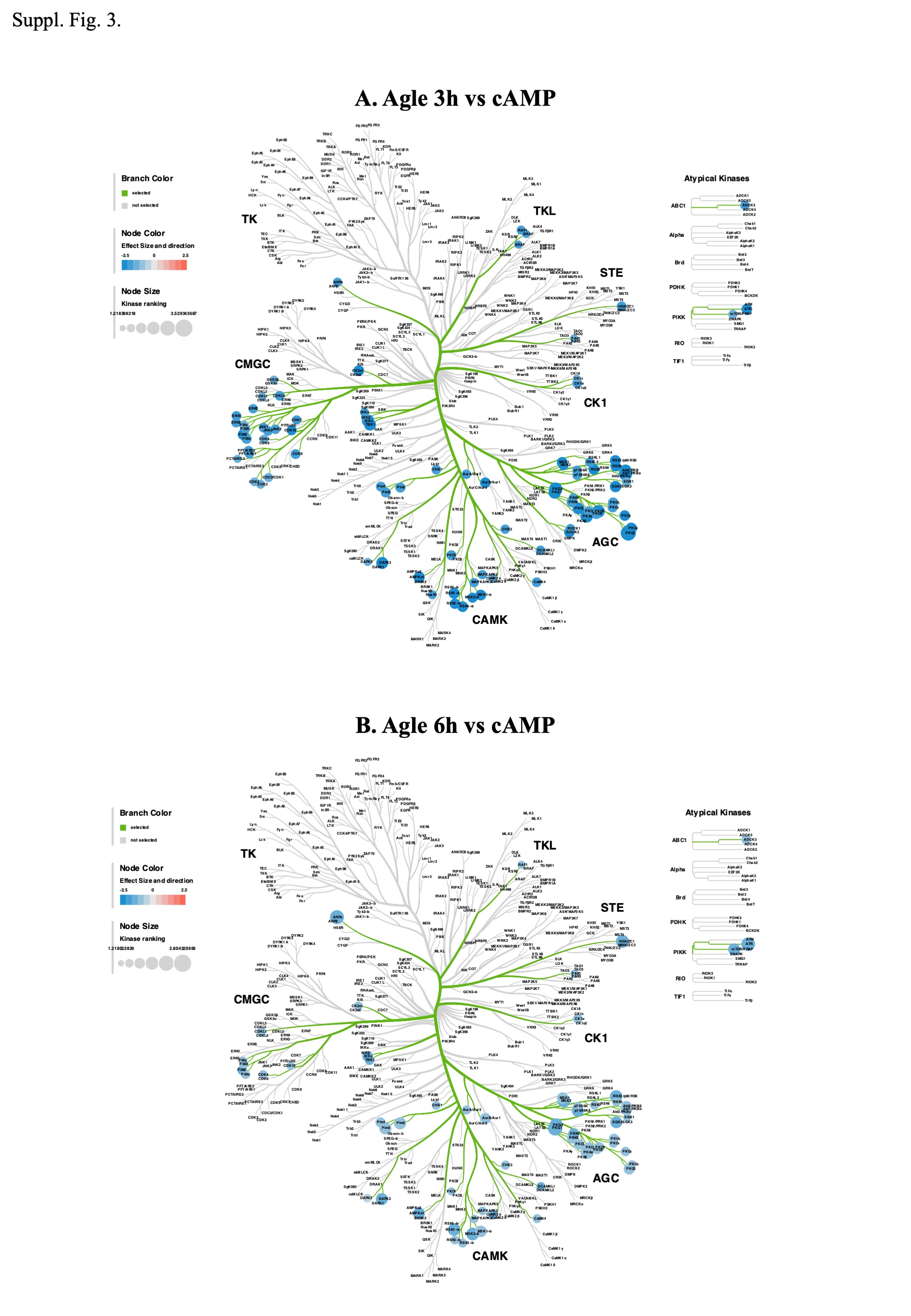

Supplement: Suppl_Fig_3_ioad170 [file suppl_fig_3_ioad170.jpeg]

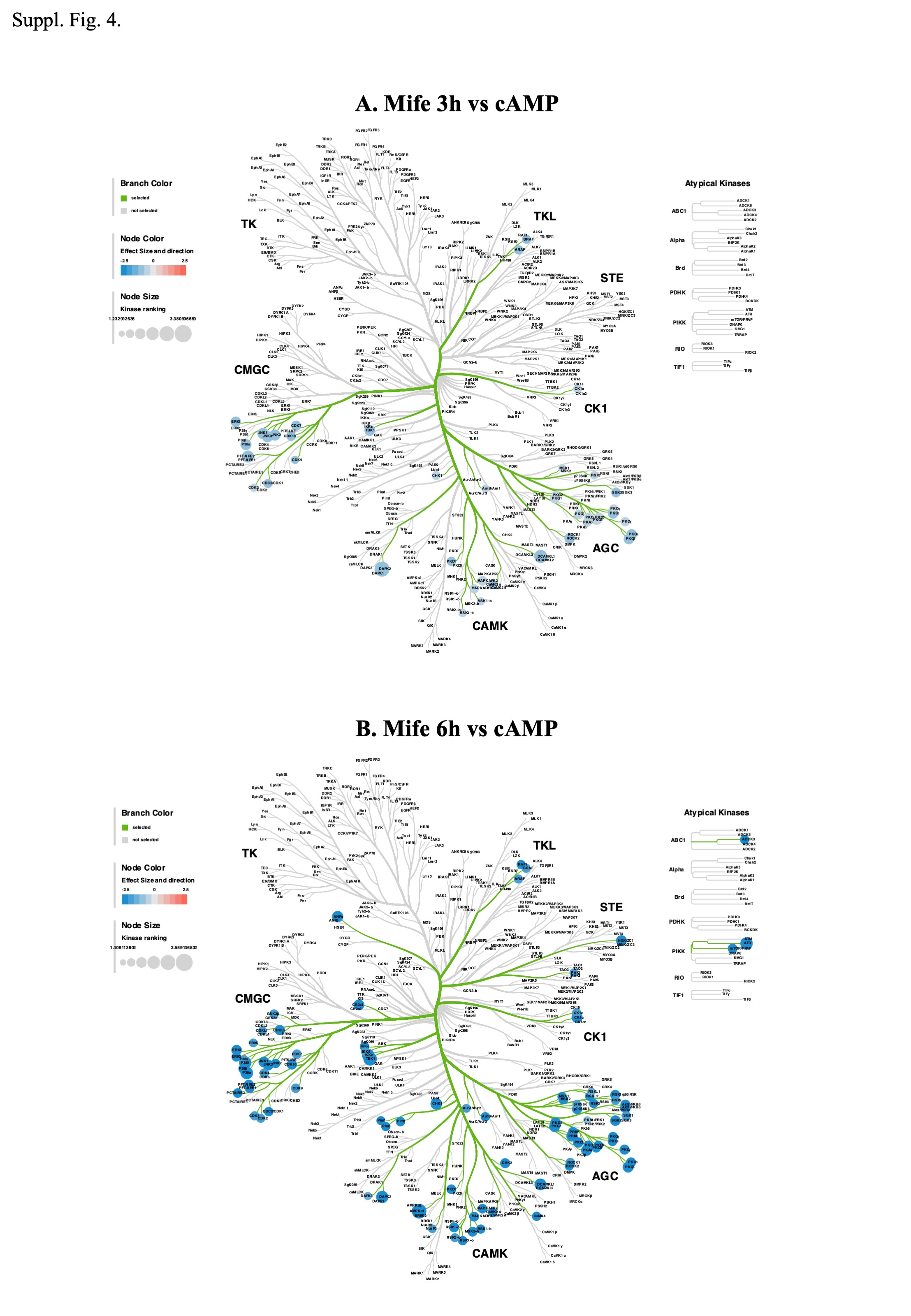

Supplement: Suppl_Fig_4_ioad170 [file suppl_fig_4_ioad170.jpeg]

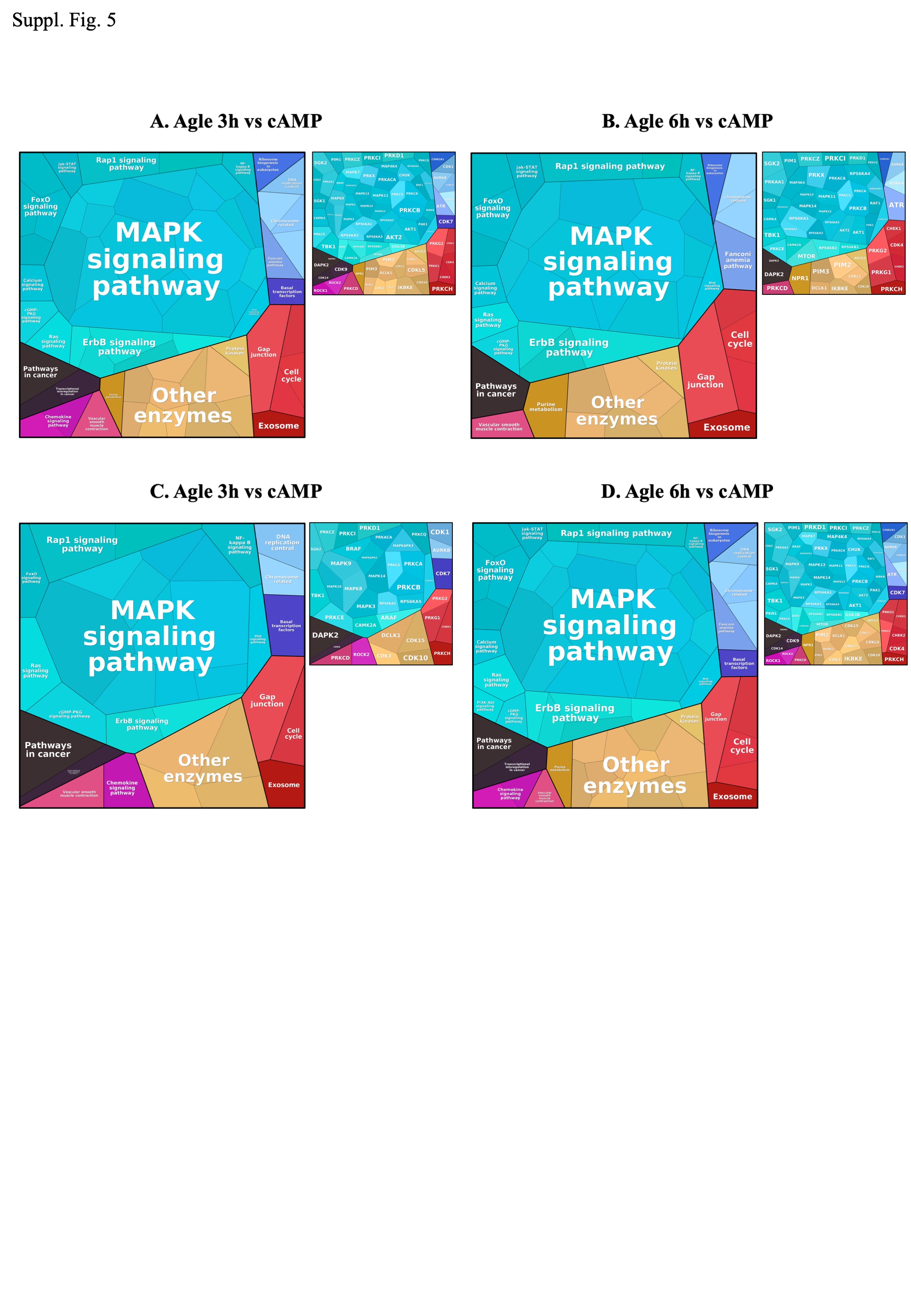

Supplement: Suppl_Fig_5_ioad170 [file suppl_fig_5_ioad170.jpeg]

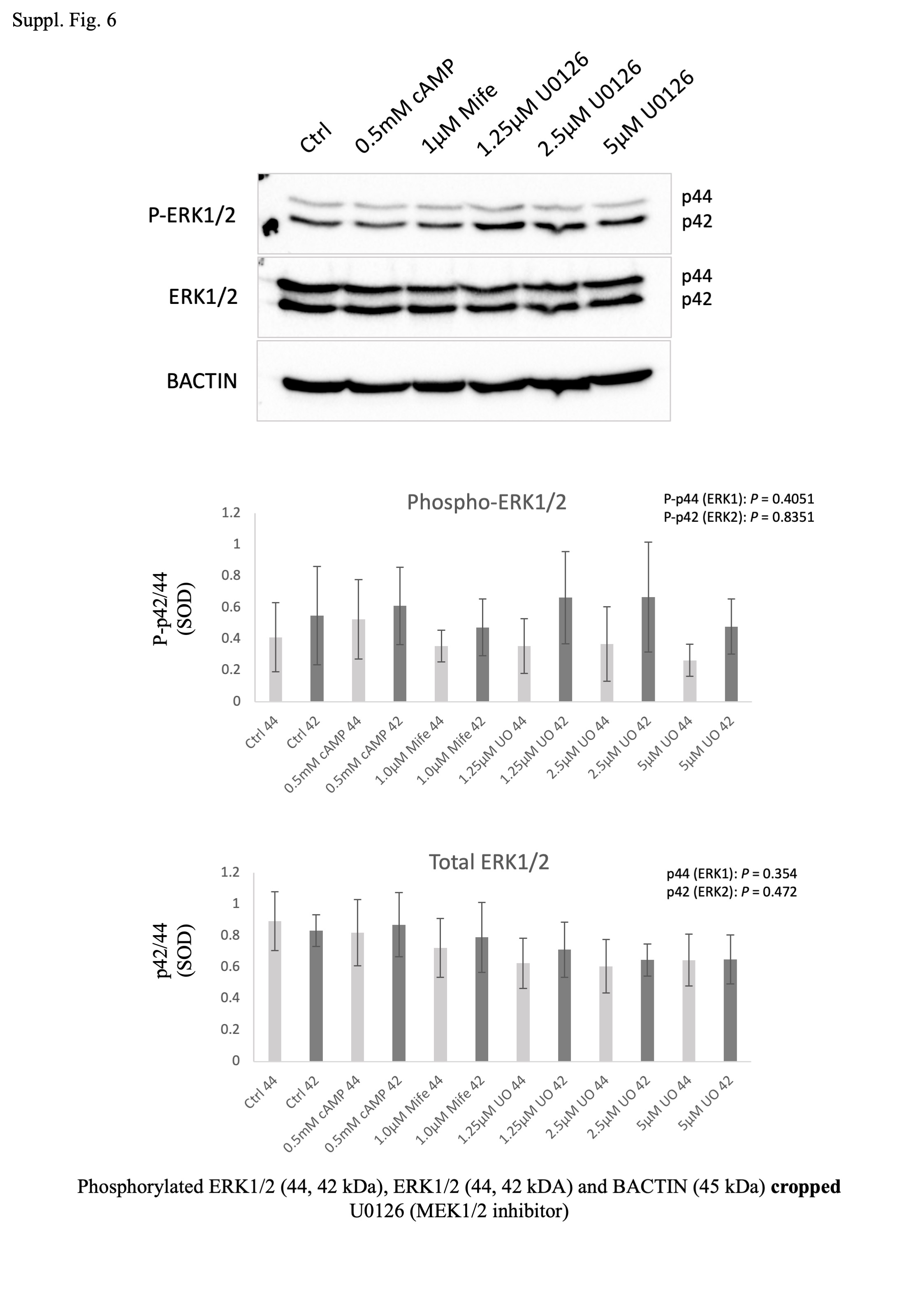

Supplement: Suppl_Fig_6_ioad170 [file suppl_fig_6_ioad170.jpeg]
