# Supplementary material for: Applying a novel kinomics approach to study decidualization and the effects of antigestagens using a canine model
Source: Biol Reprod. 2023 Dec 11;110(3):583–98. doi: 10.1093/biolre/ioad170 (PMC10941090; doi:10.1093/biolre/ioad170)
Supplement: Supplemental_Figure_legend_ioad170 [file supplemental_figure_legend_ioad170.docx]

**Supplementary Figure 1.** (A) Upstream hierarchical plot of the 85 kinases with predicted increased activity in response to cAMP-induced decidualization of DUS cells. (B) Proteomap representing the functional terms associated with the kinases significantly affected in the contrast “cAMP over Control”.

**Supplementary Figure 2.** Hierarchical plot presenting the predicted kinases with anticipated decreased activity in response to: (A) 1μM aglepristone for 3h, (B) 1μM aglepristone for 6h, (C) 1μM mifepristone for 3h, and (D) 1μM mifepristone for 6h, when compared with untreated decidualized (cAMP) DUS cells.

**Supplementary Figure 3.** Visualization in Coral plot of STKs with decreased activity in decidualized DUS cells following treatment with: (A) 1μM aglepristone for 3h or (B) 1μM aglepristone for 6h. Most affected kinases belonged to AGC, CAMK and CMGC families.

**Supplementary Figure 4.** Visualization in Coral plot of STKS with decreased activity in decidualized DUS cells following treatment with: (A) 1μM mifepristone for 3h or (B) 1μM mifepristone for 6h. Most affected kinases belonged to AGC, CAMK and CMGC families.

**Supplementary Figure 5.** Proteomap presenting the functional terms associated with the kinases significantly affected in the contrasts between antigestagen treated DUS cells and cAMP group.

**Supplementary Figure 6.** Protein expression of p44/42 (ERK1/2) and P-p44/42 (ERK1/2) in DUS cells in response to treatment with the MEKs inhibitor U0126. Representative immunoblots are presented. Relative standardized optical density (SOD) was normalized against BACTIN. Effects of treatment were evaluated with one-way ANOVA.

**Supplementary Table 1.** List of predicted STK and corresponding statistical details for all contrasts.
